# The American Medical Monthly

**Published:** 1854-03

**Authors:** 


*The American Medical Monthly.*

THIS is a new Journal of 80 pages, published under the auspices of the Faculty of the New York Medical College. Dr. Edward H. Parker, formerly of the N. H. Med. Journal, is the editor. Dr. P. is a fine writer, and well qualified for the position which he occupies.

We are not sufficiently acquainted with the wants of the Profession in N. York city to judge whether the new candidate for popular favor has been born prematurely, or whether it has been called into existence by the exigencies of the Profession. One thing is certain, the Faculty of the N. York Medical College believe that their own interest demand it, and that, of course, is a sufficient reason why it should be published. We do not object to any body's name, but we cannot avoid the thought that "*The New York Medical Monthly*" would be more appropriate. From the seven Professors whose names appear on the title page, all interested in one of the medical schools of the city of New York, we should hardly expect a national journal. This conviction is forced upon us, notwithstanding the claims put forth in their salutatory, that, "In our enterprise we know no East, no West, no North, no South. We seek to render the Journal subservient to the interest and elevation of the Profession throughout America."

The numbers of this work received are creditable to the editors and publishers. J.

